# Supplementary material for: Assessing the professional quality of life in the context of pediatric care
Source: PeerJ. 2024 Mar 15;12:e17120. doi: 10.7717/peerj.17120 (PMC10946390; doi:10.7717/peerj.17120)
Supplement: Supplemental Information 2 [file peerj-12-17120-s002.pdf]

Cover Letter / consent of Questionnaire

Dear Participants:

You are respectfully invited to participate in a scientific research study titled **"Assessing the Professional Quality of Life in the context of pediatric care".** The goal of this research is to investigate the impact of compassion satisfaction and compassion fatigue. The findings of this study will contribute to a better understanding of the compassion fatigue level and compassion satisfaction level of pediatric nurses. **Your participation in this study is vital, and we appreciate your consideration.** If you choose to participate, you will be asked to complete an online survey using Google Forms. It is estimated that the survey will take approximately 10-15 minutes to complete. **Your informed consent will be considered given if you decide to complete the survey as a participant voluntarily. This meant they agreed to participate in the study before proceeding to answer the questionnaire. Each participant could only use the link once and had the choice to select either the "I agree" or "I do not agree" checkbox. Rest assured, no personally identifying information will be collected, and your responses will be anonymous. Your participation in this study is completely voluntary, without any penalties or loss of benefits if you choose not to participate or withdraw at any time.** Additionally, there are no associated costs for participation. This research study has been reviewed and approved by the Institutional Review Board (IRB) of King Saud University, supervised by Dr. Manal Alharbi

Thank you for considering participating in this research study. By completing the survey, you are providing valuable insights that will contribute to the advancement of knowledge in the field of pediatric nursing. If you have any questions or concerns regarding this study, you may contact the **Researchers: Dr. Manal F. Alharbi & Ms. Aljawharah M. Alkhamshi**

Email: maalwahbi@ksu.edu.sa

Part one :

**1. Social demographic information**
1.1. You age:<br>………………………….. years (only number)
1.2. What is your nationality
Saudi
Non saudi
1.3. Are you?
Man
Women
1.4. Social state:
Married with kids
Married without kids
Not married with kids
Not married
1.5. If you have kids how many are, they?<br>…………….. Kids (only number)
**2. Work related information**
2.1. How long you been working as pediatric nurse?<br>……………… years (only number)
2.2. Did you have experience previously in work with pediatric patient ?
Yes
No
2.3. How long your shift:
8 Hours
12 Hours
2.4. Did you work overtime?
Yes
No
2.5. If you work overtime is optional or mandatory?
No. it is mandatory
Yes. it is optional
2.6. Did you Rota changes without asking you:
Yes
No
**3. Lifestyle information**
3.1. How is your sleep pattern?
Regular
Not regular
3.2did you eating healthy balanced diet as daily routine ?

Yes
No
3.2. How often you socialize with family and friends?
Always
Often
Rarely
3.3. Did you practice any hobbies beside your work?
Yes
No
3.8.Did you smoking ?
Yes
No

Part two :

PROFESSIONAL QUALITY OF LIFE SCALE (PROQOL)

COMPASSION SATISFACTION AND COMPASSION FATIGUE
(PROQOL) VERSION 5 (2009)

When you *[help]* people you have direct contact with their lives. As you may have found, your compassion for those you *[help]* can affect you in positive and negative ways. Below are some questions about your experiences, both positive and negative, as a *[helper]*. Consider each of the following questions about you and your current work situation. Select the number that honestly reflects how frequently you experienced these things in the *last 30 days*.

1=Never	2=Rarely	3=Sometimes	4=Often	5=Very Often

_____ 1. I am happy.
_____ 2. I am preoccupied with more than one person I *[help]*.
_____ 3. I get satisfaction from being able to *[help]* people.
_____ 4. I feel connected to others.
_____ 5. I jump or am startled by unexpected sounds.
_____ 6. I feel invigorated after working with those I *[help]*.
_____ 7. I find it difficult to separate my personal life from my life as a *[helper]*.
_____ 8. I am not as productive at work because I am losing sleep over traumatic experiences of a person I *[help]*.
_____ 9. I think that I might have been affected by the traumatic stress of those I *[help]*.
_____ 10. I feel trapped by my job as a *[helper]*.
_____ 11. Because of my *[helping]*, I have felt "on edge" about various things.
_____ 12. I like my work as a *[helper]*.
_____ 13. I feel depressed because of the traumatic experiences of the people I *[help]*.
_____ 14. I feel as though I am experiencing the trauma of someone I have *[helped]*.
_____ 15. I have beliefs that sustain me.
_____ 16. I am pleased with how I am able to keep up with *[helping]* techniques and protocols.
_____ 17. I am the person I always wanted to be.
_____ 18. My work makes me feel satisfied.

19. I feel worn out because of my work as a *[helper]*.
20. I have happy thoughts and feelings about those I *[help]* and how I could help them.
21. I feel overwhelmed because my case [work] load seems endless.
22. I believe I can make a difference through my work.
23. I avoid certain activities or situations because they remind me of frightening experiences of the people I *[help]*.
24. I am proud of what I can do to *[help]*.
25. As a result of my *[helping]*, I have intrusive, frightening thoughts.
26. I feel "bogged down" by the system.
27. I have thoughts that I am a "success" as a *[helper]*.
28. I can't recall important parts of my work with trauma victims.
29. I am a very caring person.
30. I am happy that I chose to do this work.
