# Supplementary material for: Assessing the professional quality of life in the context of pediatric care
Source: PeerJ. 2024 Mar 15;12:e17120. doi: 10.7717/peerj.17120 (PMC10946390; doi:10.7717/peerj.17120)
Supplement: Supplemental Information 3 [file peerj-12-17120-s003.pdf]

STROBE Statement—Checklist of items that should be included in reports of ***cross-sectional studies***

	Item No	Recommendation
**Title and abstract**	1	(*a*) done
		(*b*) done
**Introduction**		
Background/rationale	2	Done, pages 3-7.
Objectives	3	Done, page. 7.
**Methods**		
Study design	4	Done, page 8
Setting	5	Done, pages 8
Participants	6	Done, pages 8-9
Variables	7	Done, pages 9.
Data sources/ measurement	8*	Done, pages 9
Bias	9	Done, page 8
Study size	10	Done, pages 8.
Quantitative variables	11	Done, page 10
Statistical methods	12	Done, page 10
		Done, page 10
		Done, page 10
		Done, page 10
		Done, page 10
**Results**		
Participants	13*	Done, page 11
		Done, page 11
		Done, page 11
Descriptive data	14*	Done, page 11
		Done, page 11
Outcome data	15*	Done, page 11
Main results	16	Done, page 11
		Done, page 11
		Done, page 11
Other analyses	17	Done, page 12
**Discussion**		
Key results	18	Done, page 12-15
Limitations	19	Done, page 16
Interpretation	20	Done, page 12-15
Generalisability	21	Done, page 16
**Other information**		
Funding	22	N/A

*Give information separately for exposed and unexposed groups.

Note: An Explanation and Elaboration article discusses each checklist item and gives methodological background and published examples of transparent reporting. The STROBE checklist is best used in conjunction with this article (freely available on the Web sites of PLoS Medicine at http://www.plosmedicine.org/, Annals of Internal Medicine at

http://www.annals.org/, and Epidemiology at http://www.epidem.com/). Information on the STROBE Initiative is available at www.strobe-statement.org.
